# 4-Benzyl­piperazin-1-ium chloride chloro­form solvate

**DOI:** 10.1107/S1600536808022587

**Published:** 2008-07-23

**Authors:** Mihai G. Nema, Richard A. Varga, Cristian Silvestru, Hans J. Breunig

**Affiliations:** a Faculty of Chemistry and Chemical Engineering, Babes-Bolyai University, 11 Arany Janos Street, RO-400028, Cluj Napoca, Romania; b Institut für Anorganische und Physikalische Chemie, Universität Bremen, D-28334 Bremen, Germany

## Abstract

The ions of the title chloro­form-solvated salt, C_11_H_17_N_2_^+^·Cl^−^·CHCl_3_, are linked by a strong N—H⋯Cl hydrogen bond; the solvent mol­ecule also inter­acts with the chloride ion through a C—H⋯Cl hydrogen bond. Additionally, neighboring cations form weak hydrogen bonds to the anion, resulting in a supra­molecular ribbon that runs along the *a* axis.

## Related literature

For related literature, see Albinati *et al.* (1980 ▶); Antolini *et al.* (1981 ▶, 1982 ▶); Osa *et al.* (2002 ▶); Tanaka *et al.* (2005 ▶).
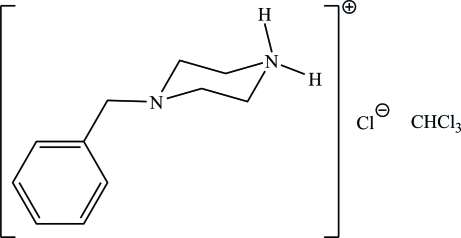


## Experimental

### 

#### Crystal data

C_11_H_17_N_2_^+^·Cl^−^·CHCl_3_
*M* *_r_* = 332.08
Triclinic, 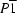

*a* = 5.6053 (4) Å
*b* = 9.4889 (9) Å
*c* = 15.303 (2) Å
α = 100.980 (8)°
β = 90.957 (7)°
γ = 93.219 (7)°
*V* = 797.51 (15) Å^3^
*Z* = 2
Mo *K*α radiation
μ = 0.73 mm^−1^
*T* = 173 (2) K
0.40 × 0.32 × 0.30 mm

#### Data collection

Siemens P4 diffractometer
Absorption correction: none
4013 measured reflections
2967 independent reflections
2811 reflections with *I* > 2σ(*I*)
*R*_int_ = 0.015
3 standard reflections every 197 reflections intensity decay: none

#### Refinement

*R*[*F* ^2^ > 2σ(*F* ^2^)] = 0.047
*wR*(*F* ^2^) = 0.128
*S* = 1.07
2967 reflections
175 parameters
2 restraints
H atoms treated by a mixture of independent and constrained refinement
Δρ_max_ = 0.56 e Å^−3^
Δρ_min_ = −0.72 e Å^−3^

### 

Data collection: *XSCANS* (Siemens, 1994 ▶); cell refinement: *XSCANS*; data reduction: *XSCANS*; program(s) used to solve structure: *SHELXS97* (Sheldrick, 2008 ▶); program(s) used to refine structure: *SHELXL97* (Sheldrick, 2008 ▶); molecular graphics: *DIAMOND* (Brandenburg & Putz, 2006 ▶); software used to prepare material for publication: *publCIF* (Westrip, 2008 ▶).

## Supplementary Material

Crystal structure: contains datablocks I, global. DOI: 10.1107/S1600536808022587/ng2473sup1.cif

Structure factors: contains datablocks I. DOI: 10.1107/S1600536808022587/ng2473Isup2.hkl

Additional supplementary materials:  crystallographic information; 3D view; checkCIF report

## Figures and Tables

**Table 1:** Hydrogen-bond geometry (Å, °)

*D*—H⋯*A*	*D*—H	H⋯*A*	*D*⋯*A*	*D*—H⋯*A*
N2—H1N⋯Cl1	0.85 (2)	2.30 (2)	3.140 (2)	169 (2)
C12—H12⋯Cl1	0.89 (3)	2.60 (3)	3.401 (2)	151 (2)
N2—H2N⋯Cl1^i^	0.88 (2)	2.26 (2)	3.096 (2)	159 (2)
C10—H10*A*⋯Cl1^ii^	0.97	2.74	3.684 (2)	165

Symmetry codes: (i) 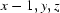; (ii) 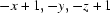.

**Fig. 1.**
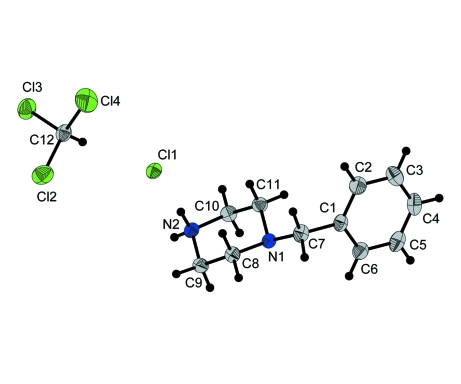
: View of the title compound showing the atom-numbering (50% probability thermal ellipsoids).

**Fig. 2.**
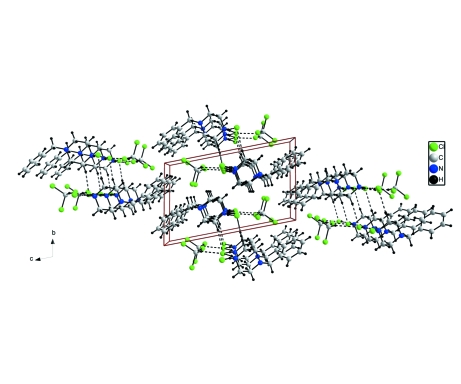
: Crystal packing of the title compound showing the supramolecular arrays (hydrogen bonds as dashes lines).

## supplementary crystallographic information

### Comment

Derivatives of piperazine are useful compounds because of their biological activity (Osa *et al.*, 2002). Trimetazidine is a clinically antianginal agent (Tanaka *et al.*, 2005). A compound of the type (NBzpipzH_2_)_2_CuCl_6_ (NBzpipzH_2_ = *N*-benzylpiperazinium dication) was reported (Antolini *et al.*, 1982) and the preparation of mercury(III) compounds (NbzpipzH)Hg_2_*X*_5_ (NbzpipzH = *N*-benzylpiperazinium monocation; *X* = Cl, Br) is known (Albinati, *et al.*, 1980, Antolini *et al.*, 1981).

The title compound (Fig.1) is formed by C_11_H_17_N_2_^+^ cation and Cl^-^ anion connected through a strong N—H···Cl^-^ hydrogen bond [H1N···Cl1 = 2.30 (2) Å, N2—H1N···Cl1 = 169 (2)°] and crystallizes with a CHCl_3_ molecule bonded to the Cl^-^ through a hydrogen bond [H12···Cl1 = 2.60 (3) Å, C12—H12···Cl1 = 151 (2)°]. Intermolecular hydrogen bonds link the Cl^-^ anion to two aditional cations (Table 1) resulting in a double chain-like supramolecular arrangement along the *a* axis. In crystal there are no interactions between the chains (Fig.2).

### Experimental

The compound was obtained as a by-product of the reaction between [2-{HN(CH_2_CH_2_)_2_NCH_2_}C_6_H_4_]Li and BiCl_3_. Crystals were grown by slow difusion from chloroform / n-hexane (1:5).

^1^H NMR (CDCl_3_, 200 MHz, 291 K): δ 2.74 (4*H*, *m*, N—CH_2_—CH_2_—N), 3.20 (4*H*, *m*, N—CH_2_—CH_2_—N), 3.55 (2*H*, *s*, C_6_H_5_—CH_2_—N), 7.29 (5*H*, *m*, C_6_H_5_), 8.90 (2*H*, *s*, br, NH_2_). ^13^C NMR (CDCl_3_, 50 MHz, 291 K): δ 43.59 (*s*, N—CH_2_—CH_2_—N), 49.47 (*s*, N—CH_2_—CH_2_—N), 62.39 (*s*, C_6_H_5_—CH_2_—N), 127.53 (*s*, C-p), 128.45 (*s*, C-m), 128.95 (*s*, C-o), 136.89 (*s*, C-i).

### Refinement

All hydrogen atoms were placed in calculated positions using a riding model, with C—H = 0.93–0.97 Å and with *U*_iso_= 1.2*U*_eq_ (C) for aryl H and *U*_iso_= 1.5*U*_eq_ (C) for the rest. The hydrogen H1N, H2N and H12 atoms bonded to N2 and C12, respectively, were found in a difference map and refined with a restrained N—H distance of 0.85 (2) and 0.88 (2) Å, and C—H distance of 0.89 (3) Å, respectively.

### Crystal data

**Table:**

C_11_H_17_N_2_^+^·Cl^–^·CHCl_3_	*Z* = 2
*M**_r_* = 332.08	*F*_000_ = 344
Triclinic, *P*1	*D*_x_ = 1.383 Mg m^−^^3^
Hall symbol: -P 1	Mo *K*α radiation λ = 0.71073 Å
*a* = 5.6053 (4) Å	Cell parameters from 44 reflections
*b* = 9.4889 (9) Å	θ = 8.5–25.1º
*c* = 15.303 (2) Å	µ = 0.73 mm^−^^1^
α = 100.980 (8)º	*T* = 173 (2) K
β = 90.957 (7)º	Block, colorless
γ = 93.219 (7)º	0.40 × 0.32 × 0.30 mm
*V* = 797.51 (15) Å^3^	

### Data collection

**Table:**

Siemens P4 diffractometer	*R*_int_ = 0.015
Radiation source: sealed tube	θ_max_ = 25.5º
Monochromator: graphite	θ_min_ = 2.7º
*T* = 173(2) K	*h* = −6→2
2θ–/ω scans	*k* = −11→11
Absorption correction: none	*l* = −18→18
4013 measured reflections	3 standard reflections
2967 independent reflections	every 197 reflections
2811 reflections with *I* > 2σ(*I*)	intensity decay: none

### Refinement

**Table:**

Refinement on *F*^2^	Secondary atom site location: difference Fourier map
Least-squares matrix: full	Hydrogen site location: inferred from neighbouring sites
*R*[*F*^2^ > 2σ(*F*^2^)] = 0.047	H atoms treated by a mixture of independent and constrained refinement
*wR*(*F*^2^) = 0.128	*w* = 1/[σ^2^(*F*_o_^2^) + (0.0804*P*)^2^ + 0.5046*P*] where *P* = (*F*_o_^2^ + 2*F*_c_^2^)/3
*S* = 1.07	(Δ/σ)_max_ < 0.001
2967 reflections	Δρ_max_ = 0.56 e Å^−^^3^
175 parameters	Δρ_min_ = −0.72 e Å^−^^3^
2 restraints	Extinction correction: none
Primary atom site location: structure-invariant direct methods	

### Special details

**Table:**

Geometry. All e.s.d.'s (except the e.s.d. in the dihedral angle between two l.s. planes) are estimated using the full covariance matrix. The cell e.s.d.'s are taken into account individually in the estimation of e.s.d.'s in distances, angles and torsion angles; correlations between e.s.d.'s in cell parameters are only used when they are defined by crystal symmetry. An approximate (isotropic) treatment of cell e.s.d.'s is used for estimating e.s.d.'s involving l.s. planes.
Refinement. Refinement of *F*^2^ against ALL reflections. The weighted *R*-factor *wR* and goodness of fit *S* are based on *F*^2^, conventional *R*-factors *R* are based on *F*, with *F* set to zero for negative *F*^2^. The threshold expression of *F*^2^ > σ(*F*^2^) is used only for calculating *R*-factors(gt) *etc*. and is not relevant to the choice of reflections for refinement. *R*-factors based on *F*^2^ are statistically about twice as large as those based on *F*, and *R*- factors based on ALL data will be even larger.

### Fractional atomic coordinates and isotropic or equivalent isotropic displacement parameters (Å^2^)

**Table:**

	*x*	*y*	*z*	*U*_iso_*/*U*_eq_	
Cl4	1.23828 (12)	0.13191 (10)	0.29039 (5)	0.0610 (2)	
C12	0.9555 (4)	0.1520 (2)	0.24518 (15)	0.0300 (5)	
Cl1	0.70662 (8)	0.21020 (5)	0.44910 (3)	0.02735 (17)	
Cl2	0.94353 (13)	0.32245 (6)	0.21628 (5)	0.0472 (2)	
Cl3	0.87877 (13)	0.01645 (7)	0.15238 (4)	0.0450 (2)	
N1	0.3777 (3)	0.37270 (18)	0.71793 (11)	0.0228 (4)	
N2	0.2108 (3)	0.25651 (19)	0.53939 (12)	0.0251 (4)	
C2	0.6514 (4)	0.2552 (3)	0.89764 (16)	0.0353 (5)	
H2	0.7872	0.2308	0.8654	0.042*	
C6	0.2964 (4)	0.3838 (3)	0.91871 (15)	0.0324 (5)	
H6	0.1924	0.4466	0.9010	0.039*	
C11	0.3743 (4)	0.2181 (2)	0.68279 (14)	0.0275 (4)	
H11A	0.3486	0.1659	0.7308	0.033*	
H11B	0.5274	0.1940	0.6572	0.033*	
C1	0.5004 (4)	0.3496 (2)	0.87052 (13)	0.0264 (4)	
C8	0.4281 (4)	0.4505 (2)	0.64568 (14)	0.0260 (4)	
H8A	0.5804	0.4242	0.6202	0.031*	
H8B	0.4386	0.5531	0.6690	0.031*	
C9	0.2325 (4)	0.4145 (2)	0.57441 (14)	0.0273 (4)	
H9A	0.0819	0.4460	0.5991	0.033*	
H9B	0.2689	0.4647	0.5262	0.033*	
C5	0.2475 (4)	0.3245 (3)	0.99313 (16)	0.0380 (5)	
H5	0.1106	0.3477	1.0249	0.046*	
C10	0.1780 (4)	0.1739 (2)	0.61243 (14)	0.0280 (4)	
H10A	0.1808	0.0718	0.5884	0.034*	
H10B	0.0239	0.1917	0.6389	0.034*	
C4	0.4008 (5)	0.2314 (3)	1.02029 (15)	0.0389 (6)	
H4	0.3681	0.1925	1.0705	0.047*	
C7	0.5559 (4)	0.4164 (2)	0.79057 (14)	0.0286 (5)	
H7A	0.5634	0.5203	0.8085	0.034*	
H7B	0.7117	0.3888	0.7691	0.034*	
C3	0.6033 (5)	0.1962 (3)	0.97243 (17)	0.0415 (6)	
H3	0.7071	0.1332	0.9902	0.050*	
H1N	0.339 (4)	0.232 (3)	0.5127 (17)	0.035 (7)*	
H2N	0.089 (4)	0.235 (3)	0.5012 (16)	0.039 (7)*	
H12	0.848 (5)	0.145 (3)	0.2865 (19)	0.039 (7)*	

### Atomic displacement parameters (Å^2^)

**Table:**

	*U*^11^	*U*^22^	*U*^33^	*U*^12^	*U*^13^	*U*^23^
Cl4	0.0309 (4)	0.0937 (6)	0.0588 (5)	0.0100 (3)	−0.0096 (3)	0.0144 (4)
C12	0.0240 (10)	0.0381 (12)	0.0271 (10)	0.0006 (9)	0.0046 (9)	0.0044 (9)
Cl1	0.0176 (3)	0.0367 (3)	0.0265 (3)	0.0034 (2)	0.00211 (19)	0.0023 (2)
Cl2	0.0565 (4)	0.0326 (3)	0.0527 (4)	0.0009 (3)	0.0136 (3)	0.0079 (3)
Cl3	0.0589 (4)	0.0362 (3)	0.0360 (3)	−0.0047 (3)	−0.0030 (3)	−0.0007 (2)
N1	0.0214 (8)	0.0232 (8)	0.0230 (8)	−0.0007 (6)	0.0010 (7)	0.0032 (6)
N2	0.0167 (8)	0.0323 (9)	0.0248 (9)	0.0026 (7)	0.0004 (7)	0.0014 (7)
C2	0.0256 (11)	0.0460 (13)	0.0335 (12)	0.0042 (9)	−0.0001 (9)	0.0053 (10)
C6	0.0274 (11)	0.0396 (12)	0.0286 (11)	0.0031 (9)	−0.0004 (9)	0.0020 (9)
C11	0.0290 (11)	0.0248 (10)	0.0283 (10)	0.0021 (8)	−0.0002 (8)	0.0043 (8)
C1	0.0231 (10)	0.0306 (10)	0.0226 (10)	−0.0044 (8)	−0.0037 (8)	0.0001 (8)
C8	0.0254 (10)	0.0253 (10)	0.0272 (10)	−0.0021 (8)	0.0027 (8)	0.0056 (8)
C9	0.0264 (10)	0.0274 (10)	0.0291 (10)	0.0035 (8)	0.0008 (8)	0.0074 (8)
C5	0.0335 (12)	0.0491 (14)	0.0282 (11)	−0.0033 (10)	0.0062 (9)	0.0003 (10)
C10	0.0268 (10)	0.0250 (10)	0.0312 (11)	−0.0029 (8)	0.0000 (8)	0.0040 (8)
C4	0.0443 (14)	0.0468 (13)	0.0246 (10)	−0.0096 (11)	−0.0027 (10)	0.0082 (10)
C7	0.0230 (10)	0.0333 (11)	0.0273 (10)	−0.0053 (8)	−0.0017 (8)	0.0023 (9)
C3	0.0418 (14)	0.0471 (14)	0.0380 (13)	0.0035 (11)	−0.0078 (11)	0.0146 (11)

### Geometric parameters (Å, °)

**Table:**

Cl4—C12	1.753 (2)	C11—H11A	0.9700
C12—Cl3	1.755 (2)	C11—H11B	0.9700
C12—Cl2	1.761 (2)	C1—C7	1.510 (3)
C12—H12	0.89 (3)	C8—C9	1.511 (3)
N1—C11	1.462 (3)	C8—H8A	0.9700
N1—C8	1.464 (2)	C8—H8B	0.9700
N1—C7	1.465 (3)	C9—H9A	0.9700
N2—C9	1.490 (3)	C9—H9B	0.9700
N2—C10	1.491 (3)	C5—C4	1.381 (4)
N2—H1N	0.852 (17)	C5—H5	0.9300
N2—H2N	0.881 (17)	C10—H10A	0.9700
C2—C1	1.381 (3)	C10—H10B	0.9700
C2—C3	1.391 (3)	C4—C3	1.383 (4)
C2—H2	0.9300	C4—H4	0.9300
C6—C5	1.387 (3)	C7—H7A	0.9700
C6—C1	1.391 (3)	C7—H7B	0.9700
C6—H6	0.9300	C3—H3	0.9300
C11—C10	1.511 (3)		
			
Cl4—C12—Cl3	111.93 (13)	C9—C8—H8A	109.6
Cl4—C12—Cl2	110.52 (13)	N1—C8—H8B	109.6
Cl3—C12—Cl2	110.17 (12)	C9—C8—H8B	109.6
Cl4—C12—H12	108.1 (19)	H8A—C8—H8B	108.2
Cl3—C12—H12	107.5 (19)	N2—C9—C8	110.19 (16)
Cl2—C12—H12	108.5 (18)	N2—C9—H9A	109.6
C11—N1—C8	109.08 (16)	C8—C9—H9A	109.6
C11—N1—C7	111.36 (16)	N2—C9—H9B	109.6
C8—N1—C7	110.31 (16)	C8—C9—H9B	109.6
C9—N2—C10	111.57 (16)	H9A—C9—H9B	108.1
C9—N2—H1N	108.5 (19)	C4—C5—C6	120.4 (2)
C10—N2—H1N	108.6 (18)	C4—C5—H5	119.8
C9—N2—H2N	109.8 (18)	C6—C5—H5	119.8
C10—N2—H2N	109.3 (18)	N2—C10—C11	110.24 (17)
H1N—N2—H2N	109 (3)	N2—C10—H10A	109.6
C1—C2—C3	120.9 (2)	C11—C10—H10A	109.6
C1—C2—H2	119.5	N2—C10—H10B	109.6
C3—C2—H2	119.5	C11—C10—H10B	109.6
C5—C6—C1	120.3 (2)	H10A—C10—H10B	108.1
C5—C6—H6	119.8	C5—C4—C3	119.6 (2)
C1—C6—H6	119.8	C5—C4—H4	120.2
N1—C11—C10	110.42 (17)	C3—C4—H4	120.2
N1—C11—H11A	109.6	N1—C7—C1	112.66 (17)
C10—C11—H11A	109.6	N1—C7—H7A	109.1
N1—C11—H11B	109.6	C1—C7—H7A	109.1
C10—C11—H11B	109.6	N1—C7—H7B	109.1
H11A—C11—H11B	108.1	C1—C7—H7B	109.1
C2—C1—C6	118.9 (2)	H7A—C7—H7B	107.8
C2—C1—C7	120.9 (2)	C4—C3—C2	119.8 (2)
C6—C1—C7	120.3 (2)	C4—C3—H3	120.1
N1—C8—C9	110.12 (16)	C2—C3—H3	120.1
N1—C8—H8A	109.6		
			
C8—N1—C11—C10	−61.9 (2)	C1—C6—C5—C4	0.1 (4)
C7—N1—C11—C10	176.10 (16)	C9—N2—C10—C11	−53.5 (2)
C3—C2—C1—C6	−0.8 (3)	N1—C11—C10—N2	57.5 (2)
C3—C2—C1—C7	178.5 (2)	C6—C5—C4—C3	−0.5 (4)
C5—C6—C1—C2	0.5 (3)	C11—N1—C7—C1	−63.9 (2)
C5—C6—C1—C7	−178.8 (2)	C8—N1—C7—C1	174.83 (17)
C11—N1—C8—C9	62.1 (2)	C2—C1—C7—N1	115.9 (2)
C7—N1—C8—C9	−175.25 (17)	C6—C1—C7—N1	−64.7 (3)
C10—N2—C9—C8	53.8 (2)	C5—C4—C3—C2	0.2 (4)
N1—C8—C9—N2	−58.0 (2)	C1—C2—C3—C4	0.4 (4)

### Hydrogen-bond geometry (Å, °)

**Table:**

*D*—H···*A*	*D*—H	H···*A*	*D*···*A*	*D*—H···*A*
N2—H1N···Cl1	0.85 (2)	2.30 (2)	3.140 (2)	169 (2)
C12—H12···Cl1	0.89 (3)	2.60 (3)	3.401 (2)	151 (2)
N2—H2N···Cl1^i^	0.88 (2)	2.26 (2)	3.096 (2)	159 (2)
C10—H10A···Cl1^ii^	0.97	2.74	3.684 (2)	165

Symmetry codes: (i) *x*−1, *y*, *z*; (ii) −*x*+1, −*y*, −*z*+1.
